# Bronchopleural Fistula after Lobectomy for Lung Cancer: How to Manage This Life-Threatening Complication Using Both Old and Innovative Solutions

**DOI:** 10.3390/cancers16061146

**Published:** 2024-03-14

**Authors:** Antonio Mazzella, Monica Casiraghi, Clarissa Uslenghi, Riccardo Orlandi, Giorgio Lo Iacono, Luca Bertolaccini, Gianluca Maria Varano, Franco Orsi, Lorenzo Spaggiari

**Affiliations:** 1 Division of Thoracic Surgery, IEO, European Institute of Oncology IRCCS, 20141 Milan, Italy; monica.casiraghi@ieo.it (M.C.); clarissa.uslenghi@unimi.it (C.U.); riccardo.orlandi@unimi.it (R.O.); giorgio.loiacono@ieo.it (G.L.I.); luca.bertolaccini@gmail.com (L.B.); lorenzo.spaggiari@ieo.it (L.S.); 2 Division of Interventional Radiology, IEO, European Institute of Oncology IRCCS, 20141 Milan, Italy; gianluca.varano@ieo.it (G.M.V.); franco.orsi@ieo.it (F.O.); 3 Division of Oncology and Hemato-Oncology, University of Milan, 20141 Milan, Italy

**Keywords:** broncho-pleural fistula, lung cancer, lobectomy, management of broncho-pleural fistula

## Abstract

**Simple Summary:**

Bronchopleural fistula after lobectomy for lung cancer is a rare and life-threatening complication. Recognizing and managing this complication in time is often very complex for the clinicians and surgeons. Several surgical and endoscopic therapeutic approaches were described for the management of broncho-pleural fistula during the time period under study; however, the various instituted treatments depend much more on the experiences of individual centers than on a codified algorithm. Indeed, at present, there are no codified guidelines in the literature for its treatment. On the basis of our 25 years of experience, we tried to outline a diagnostic and therapeutic algorithm to help clinicians in the management of this feared complication

**Abstract:**

Backgrounds: Our goal is to evaluate the correct management of broncho-pleural fistula (BPF) after lobectomy for lung cancer. Methods: We retrospectively reviewed our 25-years’ experience and reported our strategies and our diagnostic algorithm for the management of post-lobectomy broncho-pleural fistula. Results: Five thousand one hundred and fifty (5150) patients underwent lobectomy for lung cancer in the period between 1998 and 2023. A total of 44 (0.85%) out of 5150 developed post-operative BPF. In 11 cases, BPF was solved by non-invasive treatment. In nine cases, direct surgical repair of the bronchial stump allowed BPF resolution. In 14 cases, a completion intervention was performed. In six cases, we performed open window thoracostomy (OWT) after lobectomy; in two cases, the BPF was closed by percutaneous injection of an n-butyl cyanoacrylate glue mixture. In two cases, no surgical procedure was performed because of the clinical status of the patient at the time of fistula developing. Thirty-day and ninety-day mortality from fistula onset was, respectively, 18.2% (eight patients) and 22.7% (ten patients). Thirty-day and ninety-day mortality after completion pneumonectomy (12 patients) was, respectively, 8.3% (one patient) and 16.6% (two patients). Conclusions: The correct management of BPF depends on various factors: timing of onset, size of the fistula, anatomic localization, and the general condition of the patient. In the case of failure of various initial therapeutic approaches, completion intervention or OWT could be considered.

## 1. Introduction

It is universally recognized that pulmonary anatomical resection/lobectomy represents the gold standard in the treatment of Stage I-III non-small-cell lung cancer (NSCLC); this aspect has been confirmed by the latest NCCN (National Comprehensive Cancer Network) guidelines (version 3.2002. https://www.nccn.org, guidelines, accessed on 8 March 2024).

The main complications relating to this type of surgery are also well recognized. One of the least frequent but most feared complications is represented by bronchopleural fistula (BPF), i.e., a dehiscence of the bronchial stump which can arise early (within 14 days of surgery) or late (beyond 14 days). It exposes the clean pleural space to endobronchial bacterial flora, and the resulting pleural effusion can leak into the major airway and spread to the peripheral alveolar space [1].

The onset of post-pneumonectomy BPF, the study of risk factors and, above all, the establishment of an algorithm for its correct management have been widely discussed and dealt with in the literature [2,3,4], while the same cannot be said of post-lobectomy BPF.

Similarly, much has been reported about the analysis of post-lobectomy BPF risk factors, linked to the patient’s clinical history (male sex, age > 60, diabetes mellitus, hypoalbuminemia) [1,2,3,5,6], pathological state (Stage 2–4 NSCLC, neoadjuvant radiotherapy or chemotherapy) [1,7], type of operation (sleeve lobectomy, extended bronchial resection, or residual disease at the bronchial stump after surgery) [1,8], or early complications (post-operative pneumonia, acute exacerbation of interstitial pneumonia, acute respiratory distress syndrome—ARDS) [1,5,9].

Nevertheless, there is no equivalent wealth of data in the literature regarding the onset and the management of the BPF after lobectomy; indeed, the various instituted treatments depend much more on the experiences of individual centers than on a codified algorithm. This obviously depends on the rarity of this life-threatening event, compared to pneumonectomy. The incidence rate of fistula after lobar resection in fact varies from 0.6 to 4%, even if the mortality rate ranges between 16 and 72% [10,11,12,13].

However, during the time, several surgical (chest drainage, re-intervention, re-suture of bronchial stump, completion intervention) and endoscopic (fibrin glue injection, endobronchial valves, atrial/ventricular septal defect occluders, airway stents) approaches have been described, for managing fistula after lobectomy; nevertheless, at present, there are no codified guidelines for its treatment. 

Our goal is to explore and evaluate our 25-years’ experience in the treatment of lung cancer at the European Institute of Oncology (IEO), to try and outline a diagnostic and therapeutic algorithm, in order to help clinicians in the management of this feared complication. In relation to the location, size, and timing of onset, the management of this complication can vary from less invasive methods such as simple monitoring, to more invasive ones such as pleural drainage or re-operation, to even more invasive ones such as major pulmonary resections (completion pneumonectomy) or open window thoracostomy (OWT).

## 2. Materials and Methods

### 2.1. Patients

We retrospectively reviewed, according to the Strengthening the Reporting of Observational Studies in Epidemiology (STROBE) statement, our single-center experience of the management of post-lobectomy BPF [14]. The IEO Ethical Committee approved the study (UID 4429).

Five thousand one hundred and fifty (5150) patients underwent lobectomy for lung cancer in the period between 1998 and 2023. A total of 44 (0.85%) out of 5150 developed post-operative BPF. We retrospectively reviewed peri- and post-operative outcomes and medical and operative records (Table 1). Written informed consent to undergo the procedure and the use of clinical imaging data for scientific or educational purposes, or both, was obtained from all patients before the operation.

### 2.2. Algorithm for Treatment of BPF at the European Institute of Oncology

Diagnosis of BPF was made following the evaluation of clinical parameters (fever, cough, hypoxemia), blood tests (leukocytosis and rise of C-reactive protein), and diagnostic exams (chest computed tomography scan and flexible bronchoscopy). Normally, they revealed signs of BPF associated with fluid–air collection.

**Figure 1:**
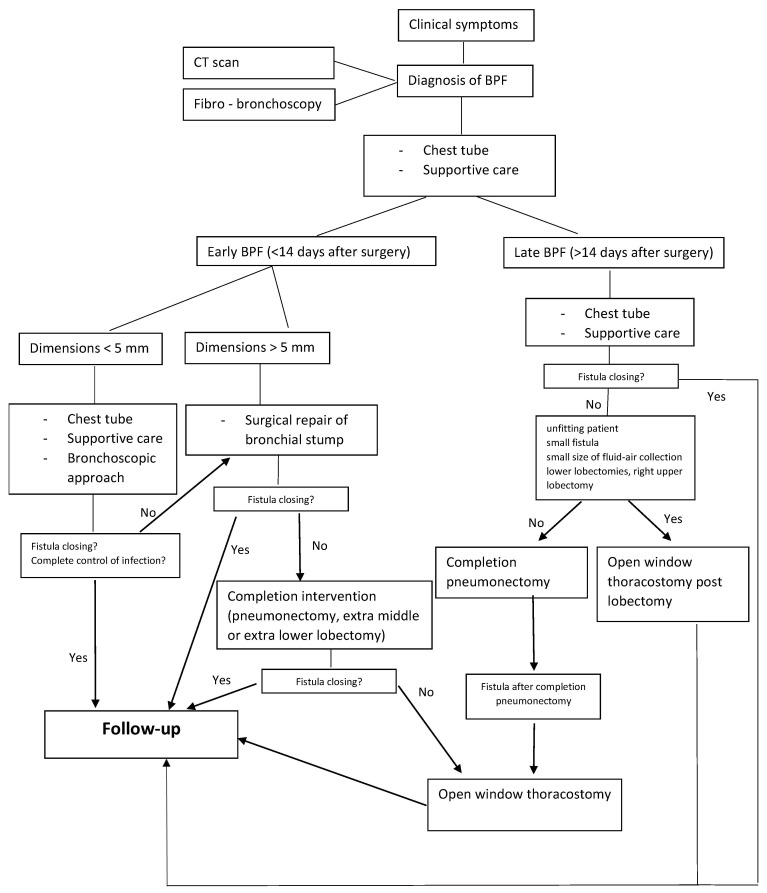
Algorithm for treatment of BPF at the European Institute of Oncology.

In the case of fluid–air collection or empyema, the first and important therapeutic option was the placement of one chest tube after diagnosis in order to evacuate the collection, to allow pleural washing, and to avoid inhalation by the remaining lung parenchyma. The second step was to assure supportive care to improve the clinical conditions of the patient; the third step was the introduction of a targeted course of antibiotic treatment, later changed, according to the results of the microbiological analysis. This was the initial treatment for all the patients (Figure 1). 

This initial approach, common to all patients, was maintained for at least 5/7 days (based on the improvement in the patient’s general condition); when the clinical conditions had stabilized, the patient was no longer critical or required intensive care, the single case was evaluated in-depth by an interdisciplinary commission (pulmonologists, intensivists, thoracic surgeons, physiotherapists) for making the following decisions.

We distinguished early (<14 days after-surgery) and late (≥14 days) BPF, on the basis of the literature and our experience [2,3,15,16].

#### 2.2.1. Early Fistula

In the case of BPF < 5 mm, an initial bronchoscopic approach (use of fibrin glue, bronchial stent, silver nitrate) was considered. Other conditions orienting towards an endoscopic approach, in addition to the size of the fistula, are the distal position of BPF or the unfit general condition of the patient to undergo surgery, 

In the case of size > 5 mm or conservative approach failure, a debridement of necrotic tissue was performed as soon as possible, with the liberation of the remaining lung, followed by a direct surgical repair of the BPF.

If possible, especially in BPF after upper lobectomy, the first step after the debridement was the isolation and the dissection of the bronchial stump; the previous suture was then removed. The fistulized bronchial stump was then sutured using non-absorbable 3-0 braided threads running through the bronchial stump wall in a U fashion and transfixing 2 separate absorbable bands 7.5 mm in width (PDS-band) to avoid bronchial wall laceration [2,3,17]. The most important thing is suturing on vital bronchial tissue, as far as possible from the fistula and from necrotic tissue. The suture is tied progressively beginning at the lateral edges, adjoining the cartilage-to-cartilage and membranous-to-membranous parts of the bronchial stump wall. The bronchial stump is then reinforced with muscle or fat flap and then buried under fibrin glue.

If a direct repair was not possible, because the suture was too close to the main bronchus or there was not enough vital tissue, a sleeve bronchial lobectomy or a completion pneumonectomy/tracheal sleeve pneumonectomy was performed. 

In the case of BPF after lower or right middle lobectomy, an extra right middle or lower lobectomy, respectively, was performed, taking care to suture the bronchus intermedius in a vital and healthy area.

#### 2.2.2. Late Fistula

In the appearance of late fistula, in cases of unsatisfactory results in terms of clinical improvement or fistula closing after the initial treatment, we adopted a more invasive treatment. In particular, when a direct surgical repair was impossible because the bronchial stump could not be separated from the surrounding fibrotic tissues, depending on the general condition of the patient (fit or unfit for major lung resection), fistula size (<5 or >5 mm), presence of empyema or not, size of fluid–air collection, and type of lobectomy, we adopted two different strategies. 

**Figure 2:**
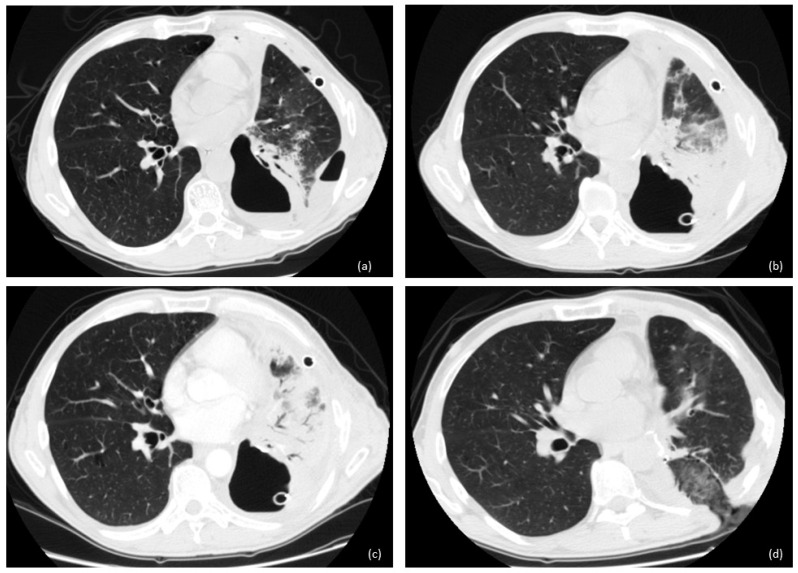
Evolution of BPF after left lower lobectomy: (**a**) empyema; (**b**,**c**) chest tube with no improvement on BPF with pneumonia of remaining lung; (**d**) open window thoracostomy with 2 gauzes in the cavity and resolution of septic and inflammatory status.

**Figure 3:**
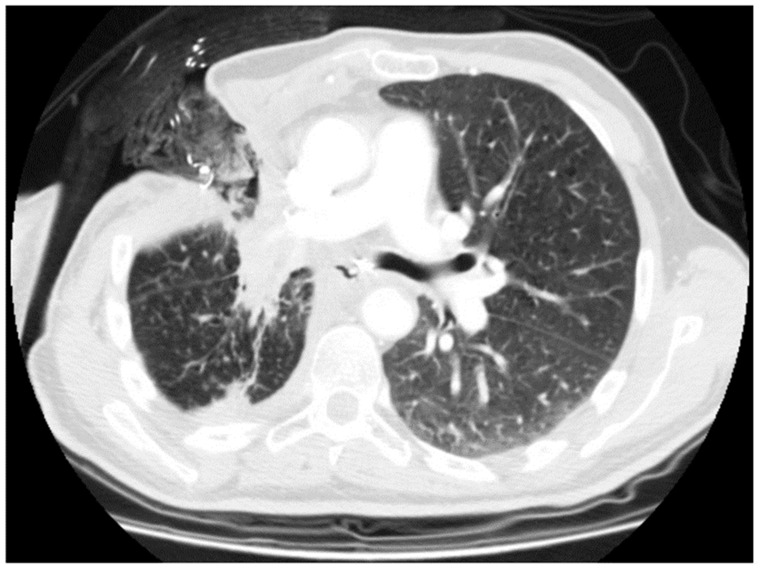
Open window thoracostomy after right upper lobectomy, after failure of bronchoscopic and minimally invasive approaches.

In the case of an unfit patient, small fistula, or small size of fluid–air collection, we opted for open window thoracostomy (OWT) (Figure 2 and Figure 3). The surgical technique of OWT has been widely discussed in our other papers [2,3].

In this case, preoperative planning with computed tomography (CT) modeling is critical for performance of the OWT. Nevertheless, in the case of fistula after left upper lobectomy, we avoided OWT, because of the proximity of the arterial to the bronchial stump. This aspect could be crucial at a later stage, during future daily OWT medications, to avoid erosion by the gauzes on the arterial tissues, or broncho-arterial fistulas. In the case of a fit patient, large fistula, or large fluid–air collection, we preferred completion pneumonectomy [18,19,20]. In the case of BPF after completion pneumonectomy, we performed a classic OWT.

### 2.3. New Therapeutic Horizons: Percutaneous Treatment of Small and Late BPF

**Figure 4:**
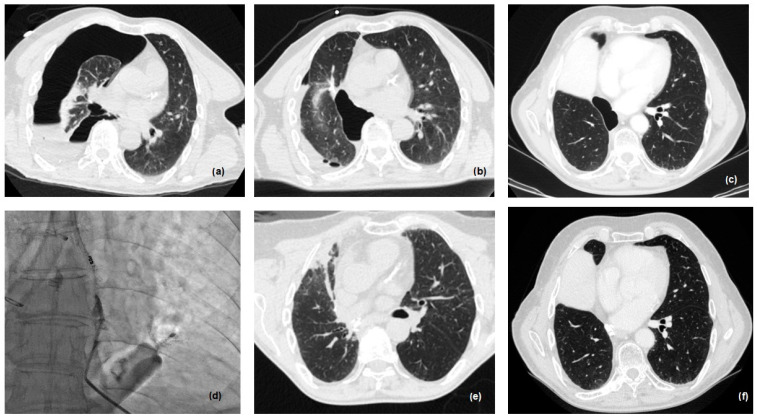
Percutaneous treatment of small and late BPF: (**a**) pneumothorax after inferior bilobectomy, 2 months after surgery; (**b**) drainage of pleural cavity; (**c**) persistence of pneumothorax after 3 months with fiberoptic diagnosis of BPF; (**d**) n-butyl cyanoacrylate (nBCA) glue injection under CT guidance, via 22-gauge Chiba needle into the postulated site of air leak/BPF; (**e**) immediate post-procedure CT scan control; (**f**) CT scan after 6 months.

In the last 2 years, in collaboration with our team in the division of interventional radiology, we have developed a new protocol, in order to treat patients presenting with small (<0.6 cm) and late fistula (Figure 4).

Indeed, percutaneous treatment of BPF after lobectomy is usually attempted in patients who are unfit or not eligible for surgery or bronchoscopic interventions, or in the case of the failure of the less invasive surgical treatment before proceeding with main surgery [21]. 

The treatment of discrete and visible fistulae is usually performed using an n-butyl cyanoacrylate (nBCA) glue injection under CT guidance. After percutaneous pleural drainage, a 20/22-gauge Chiba needle is inserted percutaneously under CT or fluoroscopic CT guidance into the postulated site of air leak and the nBCA glue mixture is subsequently injected directly into the BPF (Figure 4). The ratio of nBCA to Lipiodol in the mixture and the total amount of glue mixture injected may vary based on the size of the fistula and proceduralist preferences, (ranging in the literature from 1:3 to 4:1 and 0.2 mL to 6 mL). The use of synthetic hydrogel surgical sealant has also been described, with results similar to glue [22].

### 2.4. Patient Follow-Up

Each patient, after hospital discharge, was evaluated after one month with a chest X-ray, clinical examination, and blood tests; subsequent follow-ups consisted of tumor blood markers, blood tests, and chest/abdominal CT scan every four months for the first year and every six months for the second year.

## 3. Results

Broncho-pleural fistula occurred in 44 patients (0.85%) out of 5150 lobectomies; 11 early BPF (<14 days) and 33 late (≥14 days) BPFs occurred. The delay between surgical resection and BPF diagnosis ranged from 2 to 350 days (median 50 days).

In particular, BPF occurred after 14 right lower lobectomy (RLL) (31.9%), 2 left lower lobectomy (LLL) (4.5%), 14 right upper lobectomy (RUL) (31.9%), 4 left upper lobectomy (LUL) (9%), 5 bronchial sleeve RUL (11.3%), 2 bronchial sleeve LUL (4.5%), and 3 lower bilobectomy (6.9%).

In eight cases, BPF was treated only with chest tube, until resolution. In two cases, chest tube and bronchoscopic procedure (fibrin glue) allowed BPF resolution. Only in one case was no treatment implemented (only antibiotics therapy), and the fistula resolved spontaneously.

In nine cases, a debridement of the chest cavity and a direct surgical repair of the bronchial stump allowed BPF resolution (in one case, we performed a right upper sleeve lobectomy). 

In 14 cases, a completion intervention (12 completion pneumonectomy, 1 tracheal sleeve pneumonectomy, and 2 extra middle lobectomy) was performed. In six cases, we performed OWT after lobectomy (Figure 2 and Figure 3); in eight cases, we performed OWT after completion pneumonectomy, complicated by fistula. 

In two cases, no surgical procedure was performed because of the clinical status of the patient at the time of fistula developing (ARDS in one case, death for fatal hemoptysis at home in another case).

In the last 2 years, we introduced a new method for treating BPF, in collaboration with the division of interventional radiology. In two cases, BPF was closed by percutaneous injection of an nBCA glue mixture. The success rate in these two patients was 100%.

Thirty-day and ninety-day mortality from fistula onset was, respectively, 18.2% (eight patients) and 22.7% (ten patients).

Thirty-day and ninety-day mortality after completion pneumonectomy (12 patients) was, respectively, 8.3% (one patient) and 16.6% (two patients).

No 30-day and 90-day mortality was observed in the patients treated by OWT after lobectomy (five cases) or after completion pneumonectomy (eight cases).

In three cases out of five, OWT allowed a complete resolution of the BPF with subsequent closing of the thoracostomy by interposition of muscle (6 and 12 months later) or simple skin-edge closure (8 months later) because of the small dimensions of the cavity. In the other three patients, we did not perform closure of OWT because of the death of the patient due to cancer-related issues (two patients) or heart-attack (one patient).

## 4. Discussion

Bronchopleural fistula after lobectomy is a rare complication, with a variable onset rate between 0.6 and 4% in the literature. Even if it is rare, its mortality rate ranges from 16% to 72% [10,11,12,13]. This variability and gravity are probably linked to the rarity of this complication. At present, there is no recognized therapeutic guideline or algorithm, and its management and treatment rely on the experience of individual centers and individual surgeons.

BPF after lobectomy occurs more often in intermediate (right lower or right middle-lower lobectomy) or smaller caliber bronchi (upper lobectomy). We have not focused in-depth on the risk factors of post-lobectomy BPF that we have already listed, because this is not the topic of this paper. However, the decrease in blood supply to the bronchial stump, or the damage due to the surgical procedure, electrical burn, or devascularization of bronchial arteries, or the destruction due to local infection, have been discussed as possible causes of BPF [10,11,23,24,25]. Nevertheless, no definitive conclusion has yet been reached.

The choice of the correct therapeutic strategy for BPF is decisive in the management of this rare and life-threatening complication. Control of the septic state, improvement in the health state of patients, and prevention of pulmonary inhalation are the primary goals after BPF incidence. This phase of stabilization of the clinical general status of the patient (chest tube, antibiotics, and supportive care) is crucial for clinical and surgical management.

In our experience, this initial stabilization phase allowed a complete resolution of broncho-pleural fistula in eight cases. It is clear that the success of this conservative treatment depends on many factors: timing, dimensions, the position of the BPF, and the clinical status of the patients.

In another nine cases, it was necessary to perform a surgical debridement of the chest cavity and a direct surgical repair of the bronchial stump. This is the “gold standard” treatment in early BPF onset and its surgical technique guarantees the highest success rate. This aspect is linked to the removal of bronchial necrotic tissue and to the “new suture” on vital bronchial tissue as far as possible from the fistula, reinforced by transfixing two separate absorbable bands to avoid bronchial wall laceration [2,3,17]. In addition, the coverage of the bronchial stump with fibrin glue and muscle/fat flap allows correct blood circulation for stump healing.

In the other cases, the management appeared more complex and required the careful evaluation of various elements. 

In our experience, we have seen that OWT post-lobectomy is an appropriate and safe choice (0% of 30-day and 90-day mortality), firstly making it possible to control the septic state, stabilize and improve the clinical status of the patients, and sterilize the cavity. In addition, it is helpful for the healing of the BPF and for the obliteration of the empyematic pleural space. On the other hand, OWT requires a considerable amount of time (months or years sometimes) for the total resolution of BPF, it needs daily medication, and it affects the patient’s daily quality of life. Finally, definitive closure of the OWT requires further surgical procedures. 

A best evidence topic about the role of OWT after lung resection for lung cancer [26] reported that OWT can, in cases of failure of conservative treatments, represent a valid therapeutic tool in this group of patients. Regnard et al. [27] observed a success rate of OWT/muscle flap transposition in two out of four (50%) post-bilobectomy BPF and six out of seven (85.7%) post-lobectomy BPF, concluding that OWT is safe and effective in managing empyema post-pulmonary resection. Massera et al. [28] reported their experience with post-lobectomy (fifteen patients) or post-segmentectomy (four patients) empyema, treated by an immediate OWT followed by closure of the thoracostomy with a muscle flap within 5 months with no deaths. Sziklavari et al. [29] reported the results of 43 patients with pleural empyema, treated by VAC therapy with OWT, or mini-thoracotomy without rib spreading. All these studies considered pleural empyema with or without bronchial fistula and these results confirm the safety of the procedure but not the efficacy on late BPF. In our experience (five cases), first intention OWT made it possible to control the septic state and to completely resolve BPF. In the other three cases, the patients died before complete resolution of BPF, for completely independent reasons with respect to thoracostomy (cancer progression and one heart attack).

Completion pneumonectomy (CP) is another alternative. This is a really challenging surgical procedure, often due to the chronic inflammation sustained and exacerbated by the presence of the fistula. For this and other reasons, linked to the clinical status of this kind of patient, CP remains a highly morbid operation with a significant risk of perioperative mortality. While intraoperative mortality is practically zero, the post-operative morbidity ranges between 50 and 60% and the post-operative mortality rate is around 10–15% [19,20,30,31,32]. Our data are completely in agreement (8.3% after 30 days and 16.6% after 90 days) with those found in the literature and demonstrate how this procedure remains a high risk in the first 90 post-operative days. 

In light of these last considerations, completion pneumonectomy appears to be a sort of rescue strategy, to be implemented when all previous treatments have failed. Alternatively, it should be considered when all other techniques are unfeasible for anatomic/timing/general conditions.

In our experience, in two cases, the bronchoscopic approach allowed complete resolution of the BPF. In particular, these were two small continuity solutions, (2 mm and 3 mm) in the initial phase (20 days and 30 days after surgery); they were treated with a simple injection of fibrin glue through a flexible bronchoscope. 

The role of the bronchoscopic approach in the management of BPF (especially after pneumonectomy) has already been investigated in the literature and, day by day, this technique has gained importance, for its malleability and usefulness, as an alternative to surgery, in cases where surgical interventions are inappropriate [33,34]. Various techniques and devices have been described; in the time, they have been used: ASD/ VSD (atrial/ventricular septal defect) occluders (Amplatzer), airway stents, endobronchial valves, vascular occlusion coils, adhesive tissue, fibrin glue, and endobronchial Watanabe spigots. In some cases, this kind of minimally invasive approach could allow therapeutic success; nevertheless, the decision-making process must be multidisciplinary, and it has to consider all the different clinical and pathological conditions of the patients and of the BPF [35].

Last but not least, we must mention the role of mesenchymal stem cell transplantation technology [33,36], which is still in its embryonic stage and on which further studies are necessary to assess their long-term benefits.

It goes without saying that the correct choice of the operation timing or of the appropriate surgical technique is compulsory; our algorithm could be helpful and useful in recognizing all the phases of onset and evolution of a fistula. It also represents an important tool in the therapeutic and surgical management of all phases of the fistula.

A final aspect to take into consideration, and which we are evaluating more and more recently, is represented by the hybrid closing percutaneous approach to the fistula. In some selected cases, a simple intervention radiology procedure, via general anesthesia, allows the closure of the BPF with the use of glue and butyl-cyanoacrylate, under CT guidance. These are preliminary data (only two patients) but the success rate is complete, avoiding all post-operative problems for the patients.

It is obvious that it is a method that will have to be validated over time with further observations and evaluations, but at the moment could represent a keystone in the mini-invasive treatment of BPF.

## 5. Conclusions

The correct management of BPF depends on various factors: timing of onset, size of the fistula, anatomic localization, and the general condition of the patients. In the case of failure of the various initial therapeutic approaches, completion intervention or open window thoracostomy could be considered.

## Figures and Tables

**Table 1:** Rates of 30 and 90-day mortality from fistula onset.

Patient	Sex	Age	Original Intervention	Onset Fistula (Days)	Dimensions Fistula (mm)	Type of Operation	30-Day Mortality	90-Day Mortality	FUP
1	M	53	LUL	12	20	Surgical repair	No	No	Death (4 months)
2	F	69	SLEEVE RUL	47	10	OWT post-lob.	No	No	Death (7 months)
3	M	68	RLL	7	30	Surgical repair	No	No	Alive (110 months)
4	M	57	SLEEVE LUL	7	8	Fibrin glue	No	No	Death (10 months)
5	M	63	RUL	12	20	Surgical repair	No	Yes	Death (2 months)
6	M	56	SLEEVE RUL	7	5	Surgical repair (sleeve)	No	No	Alive (60 months)
7	M	67	RLL	45	15	CP and OWT	No	No	Death (6 months)
8	M	65	RLL	12	5	Fibrin glue	No	No	Alive (18 months)
9	M	57	RUL	150	20	OWT post-lob.	No	No	Death (13 months)
10	M	59	LLL	35	20	OWT post-lob.	No	No	Alive (24 months)
11	M	83	RUL	30	40	CP	No	Yes	Death (2 months)
12	F	45	SLEEVE RUL	23	30	CP	No	No	Alive (48 months)
13	M	44	RUL	13	15	Surgical repair	No	No	Alive (60 months)
14	M	74	RLL	14	15	Extra-middle lobectomy and OWT	No	No	Alive (132 months)
15	F	73	SLEEVE RUL	32	3	Chest tube	Yes	Yes	Death hemoptysis (0 months)
16	F	69	RLL	45	na	Chest tube	Yes	Yes	Death ARDS (0 months)
17	M	56	RUL	25	na	Chest tube	Yes	Yes	Death hemoptysis (0 months)
18	M	57	RUL	150	na	OWT post-lob.	No	No	Death (7 months)
19	M	71	Robotic LUL	90	20	OWT post-lob.	No	No	Alive (156 months)
20	M	68	RLL	350	2	Chest tube	No	No	Alive (36 months)
21	M	52	SLEEVE RUL	20	na	-	Yes	Yes	Death (0 months)
22	M	69	RLL	50	3	Surgical repair	No	No	Alive (30 months)
23	M	69	RLL	150	2	Chest tube	No	No	Alive (24 months)
24	M	74	RLL	25	2	CP	Yes	Yes	Death (1 months)
25	F	68	RLL	90	2	Chest tube	No	No	Death (72 months)
26	M	63	RUL	16	2	CP	No	No	Alive (36 months)
27	M	67	RUL	50	30	CP	No	No	Death (48 months)
28	M	64	RLL	10	20	Extra-middle lobectomy, CP, and OWT	No	No	Alive (84 months)
29	M	71	LUL	12	2	Chest tube	No	No	Death (38 months)
30	F	60	Robotic RLL	35	10	Surgical repair	No	No	Alive (60 months)
31	M	81	Robotic RUL	20	10	Surgical repair	No	No	Alive (15 months)
32	F	49	Robotic RLL	30	2	Observation	No	No	Alive (48 months)
33	M	59	LUL	60	na	-	Yes	Yes	Death (0 months)
34	M	52	RUL	350	10	CP and OWT	No	No	Death (12 months)
35	M	75	Robotic RUL	22	15	Surgical repair	No	No	Alive (48 months)
36	M	79	RUL	17	15	Chest tube	Yes	Yes	Death (0 months)
37	F	69	RLL	18	20	CP and OWT	No	No	Alive (28 months)
38	M	79	Robotic RUL	60	8	OWT post-lob.	No	No	Death (6 months)
39	M	63	Lower bilobectomy	18	25	CP and OWT	No	No	Death (4 months)
40	M	74	SLEEVE RUL	2	20	CP (tracheal sleeve pneumonectomy)	No	No	Alive (14 months)
41	F	57	Lower bilob.	47	6	Percutaneous injection of glue	No	No	Alive (18 months)
42	M	70	SLEEVE LUL	7	20	CP and OWT	No	No	Alive (6 months)
43	M	76	LLL	20	20	Extra middle lobectomy	Yes	Yes	Death (1 month)
44	M	70	Lower bilob.	12	5	Percutaneous injection of glue	No	No	Alive (7 months)

RUL: right upper lobectomy; RLL: right lower lobectomy; LUL: left upper lobectomy; LLL: left lower lobectomy; OWT: open window thoracostomy; CP: completion pneumonectomy; FUP: follow-up.

## Data Availability

Data are contained within the article.
